# Systemic Inflammation and Acute-on-Chronic Liver Failure: Too Much, Not Enough

**DOI:** 10.1155/2018/1027152

**Published:** 2018-08-01

**Authors:** Wim Laleman, Joan Claria, Schalk Van der Merwe, Richard Moreau, Jonel Trebicka

**Affiliations:** ^1^Department of Gastroenterology & Hepatology, Liver and Biliopancreatic Section, University Hospital Gasthuisberg, K.U. Leuven, Leuven, Belgium; ^2^Department of Chronic Diseases, Metabolism & Ageing (CHROMETA), Laboratory of Hepatology, KU Leuven, Leuven, Belgium; ^3^European Foundation for the Study of Chronic Liver Failure, Barcelona, Spain; ^4^Department of Biochemistry and Molecular Genetics, Hospital Clínic, IDIBAPS and CIBERehd, Barcelona, Spain; ^5^Inserm, U1149, Centre de Recherche sur l'Inflammation (CRI), UMRS1149; Université Paris Diderot-Paris 7, Département Hospitalo-Universitaire (DHU) UNITY; Service d'Hépatologie, Hôpital Beaujon, Assistance Publique-Hôpitaux de Paris; Laboratoire d'Excellence Inflamex, ComUE Sorbonne Paris Cité, Paris, France; ^6^Department of Internal Medicine I, University of Bonn, Germany; ^7^Faculty of Health Sciences, University of Southern Denmark, Odense, Denmark; ^8^Institute for Bioengineering of Catalonia, Barcelona, Spain

## Abstract

ACLF is a specific, but complex and multifactorial form of acute decompensation of cirrhosis and is characterized by an extraordinary dynamic natural course, rapidly evolving organ failure, and high short-term mortality. Dysbalanced immune function is central to its pathogenesis and outcome with an initial excessive systemic inflammatory response that drives organ failure and mortality. Later in its course, immuno-exhaustion/immunoparalysis prevails predisposing the patient to secondary infectious events and reescalation in end-organ dysfunction and mortality. The management of patients with ACLF is still poorly defined. However, as its pathophysiology is gradually being unravelled, potential therapeutic targets emerge that warrant further study such as restoring or substituting albumin via plasma exchange or via albumin dialysis and evaluating usefulness of TLR4 antagonists, modulators of gut dysbiosis (pre- or probiotics), and FXR-agonists.

## 1. Acute-on-Chronic Liver Failure and Systemic Inflammation

**Figure 1:**
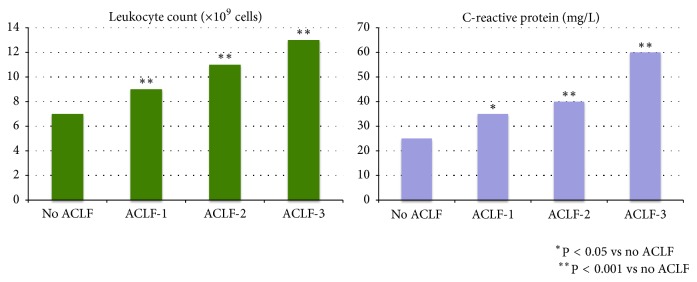
Proof of dysbalanced inflammatory response: relationship between the degree of inflammatory reaction, as estimated by the leukocyte count and C-reactive protein, and the severity of ACLF.

Acute clinical deterioration of a patient with cirrhosis remains a decisive time point in terms of medical management, since it is frequently associated with rapidly evolving multiorgan dysfunction, significant morbidity, and high short-term mortality. In the latter clinical constellation, this syndrome has been referred to as acute-on-chronic liver failure (ACLF) [1, 2]. The CANONIC study, the largest prospective multicenter study on ACLF so far with inclusion of 1343 patients admitted with acute decompensation of cirrhosis, has substantiated its relevance and clinical impact by documenting a prevalence of ACLF in this cohort of 30.9% accompanied by a high short-term mortality of 33 and 51% at 28 and 90 days, respectively (Table 1) [2]. In addition, the CANONIC study [2] and subsequent analyses [3] have exposed several premises with regard to the pathophysiology of ACLF and in particular a pivotal role for* dysregulated inflammation*. More specifically, the degree of inflammatory response, as estimated by the leukocyte count and C-reactive protein, was found to be an independent predictor of post-enrolment development of ACLF and paralleled the severity and outcome of ACLF (Figure 1). All patients with ACLF showed a high leukocyte count and C-reactive protein which in 60% of patients could be attributed to an inflammatory trigger such as bacterial infection or acute alcoholic liver injury whereas in the remaining 40% this remains undetermined at present. Together these findings suggest an altered host response to injury, both infectious and noninfectious, and immune dysfunction leading to an inappropriate inflammatory response. On the other hand, the CANONIC-trial also taught us that ACLF patients, who had earlier episodes of acute hepatic decompensation, developed a less dramatic course of ACLF with lower levels of inflammatory mediators and lower mortality rates compared to patients presenting with a first episode. This suggests that ACLF is not only associated with exaggerated inflammatory response but also with tolerance, a host defence strategy that reduces the negative impact of inflicted injury on host fitness [4].

In the next paragraphs, we will focus first on the basic mechanisms of inflammation (resistance and tolerance), secondly on the different elements contributing to this dysfunction and predominantly in the context of ACLF in patients with underlying cirrhosis (cirrhosis-associated immune dysfunction), and thirdly on additional reinforcing accomplices.

## 2. Systemic Inflammation: Resistance and Tolerance

If we focus on the healthy liver, it is to be considered as a frontline immunological organ as it balances between “resistance” and “tolerance” [5]. It acts as a gatekeeper via its unique double blood supply, the arterial blood of the hepatic artery, and the portal-venous blood delivering the products resorbed in the intestine. On the one hand, the liver maintains immune surveillance. To accomplish this role, the liver contains numerous resident antigen presenting cells strategically located to allow maximal “border control”. These involve the reticuloendothelial system (endothelial cells, Kupffer cells) and dendritic cells, which following detection participate in coordinated immune responses leading to pathogen clearance, leukocyte recruitment, and antigen presentation to lymphocytes within the unique hepatic vasculature. In addition to this local surveillance role, the liver is responsible for the bulk production of proteins involved in innate and adaptive immune responses following stimulation by proinflammatory cytokines (such as interleukin- [IL-] 6, tumor necrosis factor- [TNF-] alpha), including acute phase proteins such as C-reactive protein and lipopolysaccharide binding protein, and complement factors. Conversely, its defensive reactive role is being tightly regulated by, amongst others, high IL-10 production by Kupffer cells and Kupffer cell mediated T-cell suppression to ensure that inappropriate immune responses are not raised against nonpathogenic exogenous blood-borne molecules, such as those derived from food and conventional gut microbial antigens [5].

When a threat arises to our physical integrity, it is primarily dealt by ***“resistance”*** mechanisms. This refers to the attempts of the host immune system to “search and destroy”. Crucial in the initial resistance phase of an *infectious threat* are toll-like receptors (TLRs), which recognize distinct conserved structures in pathogens (pathogen-associated molecular patterns, PAMPs) and lead to sensing pathogen invasion, triggering innate immune responses, and priming antigen-specific adaptive immunity [6, 7]. The intracellular cascades triggered by TLR-activation lead to transcription factor activation (e.g., nuclear factor NF-*κβ*, AP-1) and subsequent transcriptional activation of hundreds of inflammatory mediator genes coding, for instance, for cytokines (i.e., TNF-*α*, IL-6, IL-1*β*, or type 1 interferons), which further shape the immune response and the elimination of bacteria and infected cells. Safeguarding the host from invading pathogens is an intricate task that requires cooperation between different pattern recognition receptors (PRRs). While responses to extracellular PAMPs are mainly mediated by membrane bound receptors such as TLRs, other cytosolic receptors (the nucleotide-binding oligomerization domain- (NOD-) like receptor, NLRs) are specialized for detection of PAMPs that reach the cytosol or intracellular organelles [7]. Several members of the NLR gene family are involved in the assembly of macromolecular protein complexes termed “inflammasomes” that lead to the activation of the inflammatory cysteine protease, caspase-1 (also known as interleukin-1 converting enzyme or ICE). Caspase-1 in turn cleaves pro-IL-1*β* or pro-IL-18, resulting in secretion of the mature and active forms of these cytokines [8].

In *noninfectious threats* due to acute tissue necrosis or immune-inflicted damage (such as fulminant hepatitis or acute alcoholic hepatitis), necrotic cells release damage/danger-associated molecular patterns (DAMPs), consisting of denaturated nuclear or cytosolic proteins, nucleic acids, and so forth, which also interact with TLRs and other specific receptors [9] (Table 2). Therefore, the host response to infectious and noninfectious (“sterile”) injury is not substantially different. DAMPs also activate inflammasomes that process the release of IL-1*β*, which initiates the activation of cytokines, as mentioned earlier [8, 9]. However, differences in host (genetic variants in genes coding for cytokines and other regulatory factors of the innate and adaptive immune systems) and pathogen (virulence, load) factors may lead to variable intensity of immune responses and susceptibility to certain pathogens. Either way, the trade-off for an exaggerated “search and destroy” strategy is collateral damage leading to “immunopathology”, defined as the negative impact of immune defence on host fitness.

To deal with this endogenous endangered physical integrity, a 2nd mechanism is activated called “**disease tolerance**” [4]. This refers to a distinct defence strategy that decreases host susceptibility to tissue damage caused by a pathogen or local factor or by the immune response directed against them (immunopathology). Whereas direct damage caused by pathogens relates to their burden and virulence and an incompetent immune response, immunopathology correlates positively with the magnitude and duration of the immune response. Therefore an optimal immune response balances between an efficient pathogen clearance and acceptable level of immunopathology.

Although much of the knowledge regarding the mechanisms involved in tolerance remains to be elucidated, logically these would be expected to prevent, reduce, or counter inflicted damage and thus involve engagement of basal and inducible homeostatic systems (amongst others by induction of stress-response genes to tone down hypersensitivity) restoring/reducing fitness costs following infectious aggression. An example of such a counterbalancing reaction is the activation of compensatory anti-inflammatory mechanisms in order to restrain a potential overzealous proinflammatory process in patients with infectious or noninfectious systemic inflammatory response. These mechanisms concert with an adapted compartmentalized response with the aim of silencing some acute proinflammatory genes and to maintain the possible expression of certain genes involved in the anti-infectious process (and by combination thus to reduce the burden of immunopathology) [10, 11]. Enhanced release of anti-inflammatory mediators such as IL-10, IL-1 receptor antagonist (IL-1RN), and soluble TNF-*α* receptor, as well as decreased HLA-DR expression (altering antigen presentation capacity) on different antigen presenting cells amongst others, dampens the inflammatory component. In contrast to what was initially postulated, the anti-inflammatory response is no longer considered a generalized damping phenomenon sequentially following a systemic inflammatory response but rather a concomitant compartmentalized reprogramming of leukocytes leading to an oscillating balance (immune dissonance) between the two opposed forces driving outcome [11, 12]. This particular premise is substantiated by the finding that repeated exposure of in vitro murine macrophages to bacterial endotoxin/ lipopolysaccharides (LPS) led to transient silencing of proinflammatory genes (e.g., TNF-*α*, IL-6, IL-1ß, IL-12, and type 1 IFN), priming of anti-inflammatory (e.g., IL-10, transforming growth factor- (TGF-) *β*, and IL-1RN) and antimicrobial effector genes, and impairing antigen presenting capacity (via decreased expression of HLA-DR), leading to a phenomenon called “endotoxin tolerance” [12–14]. These adaptive changes are also commonly associated phenotype switch (M1 →M2) and altered substrate utilization. These findings illustrate an adaptive response in macrophages and reveal component-specific regulation of inflammation adding up to the complexity of the balancing act between resistance and tolerance.

## 3. Resistance and Tolerance in ACLF: Cirrhosis-Associated Immune Dysfunction (Figure 2)

In a cirrhotic patient, immune function becomes an even more complex and often confusing matter as it can take a rapidly interchangeable and highly fluctuating course of either “too much, or not enough” resistance/inflammation [1–3, 10, 15, 16]. Recently, this immune dysfunction syndrome in the context of cirrhosis has been referred to as cirrhosis-associated immune dysfunction [17]. It consists of two concomitant, interlinked, and seemingly opposed forces: systemic inflammation and acquired immunodeficiency. These reciprocally and dynamically drive immune-(in)competence during the course of cirrhosis. This fragile balancing act is already activated in the early stages of cirrhosis (compensated state) as the cause that drives the cirrhogenesis process primes a proinflammatory phenotype by the activation of DAMPs from injury-inflicted tissue damage which is proportional to the etiological force driving chronic liver disease (such as alcohol, HBV, and HCV). As cirrhosis progresses and thus hepatocellular injury and intrahepatic shunting via completely vascularized fibrotic septae increase, the “gate-keeper function” of the liver is hollowed out. More specifically, hepatocellular insufficiency leads to the decreased production of PRRs, acute phase proteins, albumin, complement, and so forth and therefore progressive loss of opsonization, bacterial phagocytosis, and killing, while increased shunting leads to evasion of portal and systemic bacteria to the action of the reticuloendothelial system. Both features explain why bacterial products such as endotoxins and cytokines are insufficiently cleared and further prime systemic inflammation. As the severity of cirrhosis increases, bacterial translocation from the gut (see below) amplifies. This is paralleled by increased levels of PAMPs (and subsequent TLR/NLRs activation), which instigate the activation of the hepatic innate immune system, reinforcing further hepatic injury directly and indirectly through immunopathology. Initially, the spill-over of PAMPs furthers amplifies, and sometimes infuriates, the already primed proinflammatory systemic response as witnessed in LPS-stimulated cirrhotic peripheral blood mononuclear cells (PBMCs) which showed a massive induction of proinflammatory cytokines and chemokines [13, 14, 17–23].

Later on, as circulating and intestinal populations of immune cells are more and more compromised with evolving liver damage and mechanisms like “endotoxin tolerance” (with priming of anti-inflammatory (e.g., IL-10, TGF-*β*, and IL-1RA) and impairing antigen presenting capacity (via decreased expression of HLA-DR), see earlier) become generalized, the dynamic balance switches over to a predominant immunodeficient phenotype [17–19]. One of the recently elucidated pathways in this latter context is the finding of Bernsmeier et al. [24] who showed that patients with ACLF in comparison to patients with compensated and mere acute decompensation of cirrhosis had increased numbers of MER receptor tyrosine kinase (MERTK) expressing monocytes and macrophages. MERTK negatively controls innate immune response. In ACLF, MERTK expression correlated with the severity of hepatic and extrahepatic disease and systemic inflammatory response. Moreover, in vitro MERTK-inhibitors were able to restore the production of inflammatory cytokines in response to lipopolysaccharide stimulation. Additional work in this context further highlighted the important role of immunosuppressive mononuclear CD14^+^HLA-DR^−^ myeloid-derived suppressor cells (M-MDSCs) who equally quell on antimicrobial defences in ACLF [25].

However, immune activation and deficiency can coexist. Intestinal macrophages in cirrhosis are activated due to bacterial translocation (compartmentalized immune activation while at the same time immune responses may fail systemically) [20].

While endotoxin tolerance, and an anti-inflammatory response in general, is conceived as a primarily protective mechanism, its protracted duration and outbalanced intensity have been associated with high risks of secondary infections and death [11, 12, 18, 19].

The clinical implications of this skewed homeostatic balance between resistance and tolerance in cirrhosis translate in essence in the end-organ failure that determines ACLF. Although tolerance capacity differs depending on organ (given the difference in intrinsic damage susceptibility, repair capacity, functional autonomy, and damage or malfunction sequelae), dysbalanced inflammation may eventually cause organ failure through different mechanisms. First, through the action of circulating proinflammatory mediators and membrane-shed microparticles, it causes an escalation of the portal hypertensive syndrome leading to an aggravated systemic circulatory dysfunction characterized by arterial vasodilation, impairment in cardiac function, organ hypoperfusion, and end-organ ischemia [26–30]. Second, the direct extension of systemic inflammation to organs impairs cell function and may cause necrosis and/or apoptosis. In lung and kidney, detection of TLR4 forms the direct link between increasing circulating microbial products and the subsequent proinflammatory cascade in this end-organ injury [31, 32]. A recent paper by Clària et al. [3] has documented high circulating levels of pro- and anti-inflammatory cytokines in ACLF, whose levels significantly correlated with the number of organ failures. Moreover, different profiles of cytokine response were identified depending on the type of precipitating event (like, for example, alcoholic steatohepatitis or bacterial infection).

This same study revealed that not only inflammatory markers, but also markers of oxidative stress (e.g., oxidized albumin, see below) known to drive systemic inflammation, might help to identify patients with ACLF and predict their outcome. Other clinical observations underscoring the impact of this dysbalance are, for example, the observation that in patients with increasing grade of ACLF and HRS the response to terlipressin and albumin is blunted [33]. Additionally, the exaggerated anti-inflammatory response/immune-tolerance may facilitate the appearance of bacterial infections. Indeed, in the CANONIC study cohort, 46% of patients with ACLF without bacterial infections at diagnosis of ACLF developed a bacterial infection within 4 weeks, with devastating impact on short term mortality [34].

Finally, inflammation increases the release of local procoagulant factors (including tissue factor and membrane microparticles) from the endothelial cells, inducing microthrombosis in the microcirculation of different organs [35].

In conclusion, in addition to impaired circulatory function, systemic inflammation may lead to organ failure by a direct effect of the inflammatory mediators on microvascular integrity, cell function, and death mechanisms. As such the peripheral vasodilation theory no longer exclusively explains the mechanism of organ failure but embraces the systemic inflammation hypothesis in evolving cirrhosis [36].

## 4. Other Partners in Crime

In addition to inflammation, the following factors are thought to contribute as “accomplices” in the hold-up ACLF imposes on a patient.

### 4.1. Inflammaging and Immunosenescence

Patients with ACLF in the CANONIC study were of younger age underlining that the younger patients have the stronger response and thereby are more susceptible to develop ACLF [2]. In subsequent publications using different independent cohorts for the elaboration of the CLIF-C-AD score, a measure to calculate the risk of ACLF and death, younger age seems to be associated with ACLF development [37]. Previously, such trends did not reach statistical significance, but were also described [38, 39]. However, looking on the other side, TLR expression and function declines with age, thereby leading to an inadequate response to infections [40]. But on the other side, rate and severity of infections are higher and the outcome is poorer in older patients [41].

The concepts of immunosenescence and inflammaging might render these thoughts even more complex [42, 43]. Immunosenescence, characterized by impaired adaptive and innate immune systems (from decrease in naïve T-cells, increase in memory cells, skewing of myeloids, impaired chemotaxis, and effector functions in neutrophils to defects in NK-cells and monocyte dysregulation [44]), leads also to unsustained memory response to new antigens and might increase the rate of autoimmune responses, as well as inflammaging [45]. Inflammaging is a lingering, low-grade chronic inflammation. This proinflammatory environment is mainly due to the senescence-associated secretory phenotype (SASP) of the senescent immune cells [42, 43], resembling on the one side the processes during chronic liver injury and fibrogenesis [45] and on the other side the processes in mitochondria and autophagy-inflammation-cell death axis, which are quite similar to those described for alcoholic hepatitis and NASH [46–49].

Interestingly, chronic latent viral infections such as CMV and HCV might promote immunosenescence [50, 51] and thereby predispose even younger patients to ACLF. Most importantly the immunological ageing is additionally shaped by infections, and those might tailor the inflammatory response to specific insults [45, 52–54]. Chronic latent viral infections, especially CMV, but also HCV and HIV, might promote chronic systemic inflammation and increased levels of proinflammatory cytokines (IL-6, TNF-*α*), associated with premature death [51, 52, 55–57]. The low-grade inflammation after latent viral infections also induces premature ageing, predisposing cirrhotic patients to ACLF. This is supported by the fact that reactivation of HBV is a major precipitating factor for the development of ACLF, especially in Asia [39, 58, 59]. Hepatitis E might also be an important trigger for the development of ACLF, whose role is still in discussion [39].

Moreover, inflammaging is associated with impaired production of estrogen and androgen, an impairment that is also present in cirrhosis [60, 61]. Therefore, besides age, the latent infections, mitochondria damage, and decreased sexual hormones might lead to premature immunosenescence and inflammaging in chronic liver disease and predisposing for ACLF development.

### 4.2. Albumin and Prostaglandin E_2_ (PGE_2_)

Albumin, the most abundant extracellular protein in our system and synthetized exclusively by the liver, is pivotal in maintaining colloid osmotic pressure (for about 70%) but is also endowed with other vital non-oncotic properties, such as antioxidant and scavenging activity (via its sulfhydryl-groups), binding of highly toxic reactive metal species (Cu, Ni, Co, and Fe), and transport of endogenous (such as bilirubin, endotoxin, long-chain fatty acids) and exogenous toxins via the amino-terminal NH2 [62]. Albumin has been found to be a predictor of survival both in compensated and decompensated cirrhosis and ACLF [63, 64]. Studies have shown that in patients with cirrhosis albumin is subjected to posttranscriptional modifications leading to oxidized forms of albumin with impairment of its non-oncotic biological properties and thus leading to decreased “effective” albumin concentration [60].

Emerging recent evidence links this decreased “effective” (no longer native and reduced) albumin in decompensated cirrhosis to increased circulating PGE_2_-bioavailability [65]. PGE_2_, a cyclooxygenase-derived lipid mediator, is known to play a dual role in immunity since it is a major mediator of inflammation and fever, but a potent inducer of immune suppression by depressing the effector functions of macrophages and neutrophils [66]. Increased free PGE_2_ levels, due to decreased effective binding capacity of albumin, might therefore explain the profound immunodeficiency and associated bacterial infections typical of acutely decompensated cirrhosis. Turning this paradigm around, the authors showed that treatment of five patients with acutely decompensated cirrhosis with 200 ml of 20% HSA increased serum albumin concentrations from 23 g/l to 30 g/l and reversed immunosuppression [65]. In an extended and larger sample size feasibility study of 20% HSA infusions, the same group has meanwhile confirmed that infusions to raise serum albumin above 30 g/L reversed plasma-mediated immune dysfunction [67]. However, in this study the reversal of immune dysfunction following HAS therapy appeared to be mediated by changes in the circulating levels of a novel series of anti-inflammatory and proresolving lipid mediators generated from long-chain omega-3 polyunsaturated fatty acids rather than by binding of PGE_2_ [67].

Attempts to substitute or cleanse albumin (via albumin dialysis or plasma exchange) might therefore prove interest and warrant further investigation. In favour of this premise is the recently presented ANSWER study [68]. This randomised, controlled trial of 440 patients with cirrhosis and uncomplicated ascites compared standard diuretic therapy with standard diuretic therapy plus human albumin (40 g intravenously twice weekly in the first two weeks and then once weekly). Treatment with human albumin reduced the risk of death by 38%. In addition, albumin infusions also rendered significant benefits with regard to management of ascites, complications of cirrhosis (spontaneous bacterial peritonitis (SBP), non-SBP bacterial infection, renal dysfunction, hepatorenal syndrome, and hepatic encephalopathy), quality of life, and hospital admissions.

### 4.3. Farnesoid X-Receptor (FXR)

FXR is a ligand-activated transcription factor belonging to the nuclear receptor superfamily and acts as sensor for a broad range of natural ligands with bile acids as the most potent ones, in particular chenodeoxycholic acid. Therefore, FXR is highly expressed in bile acid-handling tissues such as liver, intestine, and kidney. Upon binding of bile acids to FXR, the receptor translocates to the nucleus where it forms a heterodimer with its binding partner retinoid-X receptor (RXR) and through its DNA-binding domain directly influences the transcription of a large variety of target genes [65, 66]. Since FXR is at the crossroad of metabolic regulation, inflammation, and regeneration in normal tissue, it is driving key regulator functions.

Recent translational research has suggested a central role for defective farnesoid-X-receptor signaling in hepatic inflammation, portal hypertension, and intestinal bacterial translocation, factors which are known to promote and shape ACLF and are potentially targetable through pharmacological agonists [69–74].

### 4.4. Gut Microbiota

Intestinal dysbiosis is characterized by imbalanced quantitative and qualitative changes in the composition of the gut microbiota and is associated with alterations of metabolic activity as well as an altered distribution of its microbial members. In recent years, accumulating evidence has indicated that microbial products trigger and instigate liver inflammation and that progressive qualitative changes in the gut microbiome (autochthonous to non-autochthonous taxa abundance) accompany cirrhosis and become more severe in the setting of decompensation [75]. In a recent case-control study in patients with ACLF of diverse etiology, the severity of gut dysbiosis was found worse in ACLF than in cirrhosis (considered as “a press disturbance” implying long-term impact on an ecosystem) with only moderate impact of antibiotics on its composition [76]. Additionally, the authors found that the specific gut dysbiosis in ACLF was associated with outcome, with abundance of Pasteurellaceae as independent predictor of mortality. More specifically, network-analysis comparison showed robust correlations between specific bacterial families (Ruminococcaceae and Lachnospiraceae) and inflammatory cytokines (IL-6, TNF-*α*, IL-2) in ACLF patients, indicating that gut microbiota constitutes a major backbone in ACLF pathogenesis and perpetuation [48].

## 5. Conclusions

ACLF is a specific, but complex and multifactorial form of acute decompensation of cirrhosis and is characterized by an extraordinary dynamic natural course, rapidly evolving organ failure, and high short-term mortality. Dysbalanced inflammation is central to its pathogenesis and outcome with an initial excessive systemic inflammatory response associated that drives organ failure and mortality. Later in its course, immuno-exhaustion/immunoparalysis prevails predisposing the patient to secondary infectious events and reescalation in end-organ dysfunction and mortality.

Further studies are needed to evaluate and characterize the evolving course of systemic inflammation starting from compensated cirrhosis over mere acute decompensation to ACLF. In addition, specific systemic inflammation signatures and organ dysfunction/failure are to be assessed as are specific inflammatory prophiles per triggering event. These studies hopefully will be able to guide future management, in terms of both prevention and treatment and/or organ specific approaches, for patients with AD and ACLF. For now, as its pathophysiology is gradually being unravelled, potential therapeutic targets emerge that warrant further study such as restoring or substituting albumin via plasma exchange or via albumin dialysis and evaluating usefulness of TLR4 antagonists, modulators of gut dysbiosis (pre- or probiotics), and FXR-agonists.

## Figures and Tables

**Figure 2:**
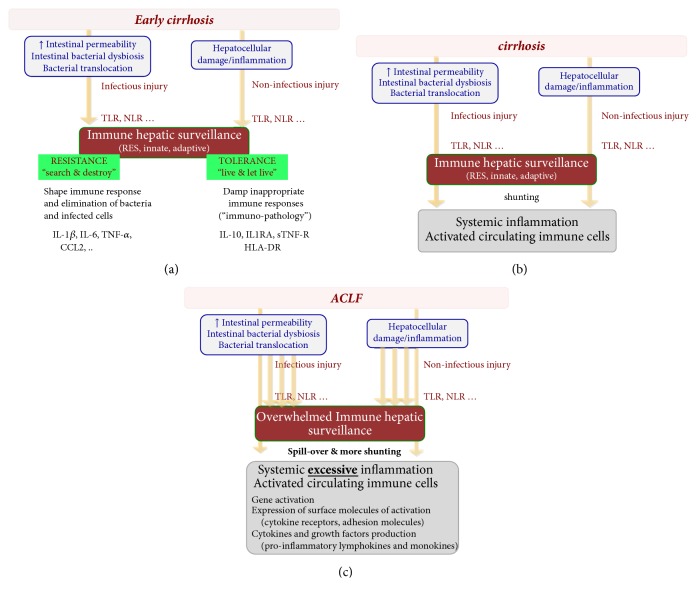
The dynamic course of immune function in evolving cirrhosis: cirrhosis-associated immune: (a) early cirrhosis sets the stage for ACLF; (b) evolving cirrhosis primes the systemic immune system and finally culminates in ACLF (c).

**Table 1:** Definition and prognosis of ACLF.

**Grade of ACLF**	**28-day mortality**	**90-day mortality**
ACLF grade 1		
(i) Single kidney failure	**22.1**%	**40.7**%
(ii) Single “non-kidney” organ failure with serum creatinine ranging from 1.5 mg/dl to 1.9 mg/dl and/or grade I or II hepatic encephalopathy		

ACLF grade 2: Presence of 2 organ failures	**32**%	**52.3**%

ACLF grade 3: Presence ≥3 organ failures	**76.7**%	**79.1**%

Minimal organ failures defined by the modified Sequential Organ Failure Assessment (SOFA) score for patients with cirrhosis:

(i) Liver: bilirubin ≥ 12mg%

(ii) Kidney: creatinine ≥ 2.0mg%

(iii) Cerebral: hepatic encephalopathy ≥ grade 3

(iv) Coagulation: INR ≥ 2.5 or platelets < 20.000 per mm^3^

(v) Circulation: need of vasopressors

(vi) Lungs: PaO/FiO2 > 100

**Table 2:** Examples of well-characterized DAMPs (danger signals or alarmins).

**DAMPs**	**Receptors**	**Outcome of receptor ligation**
Extracellular nucleotides (ATP, ADP, adenosine)	PI, P2X, and P2Y receptors (ATP, ADP); Al, A2A, A2B, and A3 receptors (adenosine)	Dendritic cell (DC) maturation, chemotaxis, secretion of cytokines (IL-1*β*, IL-18), inflammation
Extracellular heat shock proteins	CD14, CD91, scavenger receptors, TLR4, TLR2, CD40	DC maturation, cytokine induction, DC, migration to lymph nodes
Extracellular HMGB1	RAGE, TLR2, TLR4	Chemotaxis, cytokine induction, DC activation, neutrophil recruitment, inflammation, activation of immune cells
Uric acid crystals	CD14, TLR2, TLR4	DC activation, cytokine induction, neutrophil recruitment, gout induction
Laminin	Integrins	Neutrophil recruitment, chemotaxis
S100 proteins or calgranulins	RAGE	Neutrophil recruitment, chemotaxis, cytokine secretion, apoptosis
Hyaluronan	TLR2, TLR4, CD44	DC maturation, cytokine production, adjuvant activity
IL-1 family		
IL-1*α*	IL1R1 and IL1RAP	Inflammatory; promotes activation, costimulation, and secretion of cytokines and other acute-phase proteins; pyrogenic
IL-33	IL1RL1 and IL1RAP	Inducer of type 2 immune responses, activating T helper 2 (TH2) cells and mast cells; stimulates group 2 innate lymphoid cells (ILC2s), regulatory T (Treg) cells, TH1 cells, CD8+ T cells and natural killer (NK) cells.
Mitochondrial DAMPs		
mtDNA	TLR9	Proinflammatory cytokines, neutrophil chemoattraction and matrix metalloproteinase secretion, type I IFN responses 91
*N*-Formylated peptides	FPR	Neutrophil chemoattraction

## Abbreviations

ACLF: Acute-on-chronic liver failure
PRR: Pattern recognition receptor
IL: Interleukin
TNF: Tumor necrosis factor
NLR: Nucleotide-binding oligomerization domain- (NOD-) like receptor
TLR: Toll-like receptor
PAMPs: Pathogen-associated molecular patterns
DAMPs: Damage/danger-associated molecular patterns
SASP: Senescence-associated secretory phenotype
FXR: Farnesoid-X-receptor
EASL-CLIF: European Association for the Study of the Liver-Consortium on Chronic Liver Insufficiency.
